# Ethnobotanical Study of Medicinal Plants Used against COVID-19

**DOI:** 10.1155/2022/2085297

**Published:** 2022-09-15

**Authors:** Mohamed Chebaibi, Dalila Bousta, Mohammed Bourhia, Soukayna Baammi, Ahmad Mohammad Salamatullah, Hiba-Allah Nafidi, Hasnae Hoummani, Sanae Achour

**Affiliations:** ^1^Biomedical and Translational Research Laboratory, Faculty of Medicine and Pharmacy of the Fez, University of Sidi Mohamed Ben Abdellah, BP 1893, Km 22, Road of Sidi Harazem, Fez, Morocco; ^2^Laboratory of Biotechnology, Environment, Agri-Food, and Health (LBEAS), Faculty of Sciences, University Sidi-Mohamed-Ben-Abdellah (USMBA), Fez 30050, Morocco; ^3^Higher Institute of Nursing Professions and Technical Health, Laayoune 70000, Morocco; ^4^African Genome Centre (AGC), Mohammed VI Polytechnic University, Benguerir, Morocco; ^5^Department of Food Science & Nutrition, College of Food and Agricultural Sciences, King Saud University, 11 P.O. Box 2460, Riyadh 11451, Saudi Arabia; ^6^Department of Food Science, Faculty of Agricultural and Food Sciences, Laval University, Quebec 2325, QC G1V 0A6, Canada; ^7^Laboratory of Pharmacology and Toxicology, University Hospital Hassan II-Fez, Fez, Morocco

## Abstract

During the COVID-19 pandemic, the Moroccan population, like the entire population of the world, used medicinal plants to treat or cure symptoms of SARS-CoV-2. The present work was designed to identify the medicinal plants used by the Moroccan population in the prevention or treatment of COVID-19. To achieve this goal, a survey was conducted to collect data on plants along with the sociodemographic parameters of users. The outcome of this work showed that 1,263 people were interviewed with 63.5% male, aged between 18 and 82 years. Most plant users were between 20 and 40 years, which constituted 80.1% of the study population. The level of education of participants was 70.9% university and 27.6% secondary. The most useful plants were eucalyptus, cloves, lemon, and garlic. Notably, 61.9% of interviewed people used plants for preventing or treating COVID-19: 30.6% of them declared one-time use from the beginning of the pandemic, and 47.8% declared frequent daily use until recovery, while 17.4% declared single daily use. Five out of twenty-one plants used in the treatment are known for their potential toxicity, including *Artemisia herba-alba* and oleander (*Nerium oleander*). The findings of the present work could serve society by providing potential medicinal plants to control COVID-19.

## 1. Introduction

On May 27, 2020, the World Health Organization (WHO) declared the severe acute respiratory syndrome (SARS) of the current coronavirus disease 19 (COVID-19) outbreak, emerging in China at the end of 2019, as a pandemic. Since then, more than 585,568,206 confirmed cases of COVID-19, including 6,428,220 deaths across the world have been recorded, according to the website https://www.worldometers.info/coronavirus/, accessed on August 4th, 2022 [1]. Alpha, Beta, Gamma, and Delta-coronaviruses are the four genera that make up the *Coronaviridae* family, with Alpha and Beta-coronaviruses being the human pathogens. The virus that causes COVID-19, SARS-CoV-2, also known as 2019-nCoV, and severe acute respiratory syndrome (SARS) coronavirus (CoV)-2 virus, belongs to the genus Beta-coronavirus of the *Coronaviridae* family [2].

All the populations of the world were oriented towards natural products to prevent or treat infection caused by COVID-19 [3–5]. The Moroccan population is closely linked to phototherapy, which is back to several reasons, such as the richness of the country by medicinal plants (5.200 species and subspecies, and 600 species are medicinal plants), the economic situation of the Moroccan population, the illiterate and the inaccessibility of modern medicine) [6].

According to the Moroccan Ministry of Health, 1,261, 816 confirmed cases of COVID-19 and 1618, 9 deaths have been recorded since the beginning of the pandemic. As a consequence, the Moroccan population has used plants for preventing or treating this causative agent of the severe acute respiratory syndrome [7, 8].

In this context, our study aimed to collect information on medicinal plants used by the Moroccan population in the prevention or treatment of COVID-19.

## 2. Materials and Methods

### 2.1. Type and Study Area

The present work was a prospective longitudinal cohort study, which aimed to collect data by use of a structured questionnaire via Google Forms, conducted in different regions of Morocco.

### 2.2. Information Gathering

All information was collected from 10 regions in Morocco: Marrakech-Safi, Béni Mellal-Khenifra, Fez-Meknes, Casablanca-Settat, Dakhla-Oued Ed-Dahab, Drâa-Tafilalet, Laâyoune-Sakia El Hamra, Oriental, Tanger-Tetouan-Al Hoceima, and Rabat-Salé-Kénitra (Table 1).

Each questionnaire was focused on two parts; sociodemographic characteristics and ethnomedicinal data.

### 2.3. Statistical Analyses

Variables were described by use of descriptive statistics; qualitative variables were described in terms of percentage, while quantitative variables were described in terms of mean, extreme values, and standard deviation. Data entry and statistical analysis were performed by use of IBM SPSS Statistics for Windows, version 21 (IBM Corp., Armonk, NY, USA).

## 3. Results and Discussion

### 3.1. Sociodemographic Characteristics

One thousand two hundred sixty-three people were interviewed in this study (63% men vs 37% women) whose age was between 18 and 82 years. 70.9% of them have a higher education level, followed by secondary level (27.6%) and then primary level (1.5%). 41.6% of plant users had a lower monthly income of 100 USD, 24.5% between 100 and 500 USD, 24.5% between 500 and 1000 USD, and 10.5% had more than 1000 USD. 80% are located in rural areas and 27% are without medical recovery (RAMED) (Table 2).

The coronavirus responsible for COVID-19, which was identified in December 2019 in China, has infected more than 574,157,623 people worldwide and caused more than 6,401,683 deaths at the date of revising the present article. Morocco is one of the most affected countries with a high circulating potency of SARS-CoV-2.

Moroccan population has used medicinal plants for therapeutic pauses for thousands of years. Moroccans have inherited the phytotherapy knowledge from the previous generation either verbally or written, recorded history [6, 9]. Moroccan population has used plants to treat symptoms of COVID-19 as reported in earlier work [5].

Unlike several ethnobotanical studies in which illiterates and women are the majority of users of plants [10–12], in our study, mostly, educated men were the users of plants in the prevention or treatment of COVID-19.

Furthermore, the economic conditions and the low income of people influence the use of plants; people with a low income were the majority of users of plants in the treatment [10, 11, 13]. Notably, people with a low income (less than 100 USD) were the most active consumers of plants during the COVID-19 pandemic.

Plants are largely used in rural than urban areas, which can be explained by the fact that people in rural areas have more accessibility to herbal products [14]. This result is in agreement with our study, wherein 80% of our population are living in rural areas.

### 3.2. Ethnobotanical Data

#### 3.2.1. Plants Used

In total, 21 plants belonging to twelve botanical families have been used to treat or prevent COVID-19. The mostly used plants are eucalyptus, cloves (*Syzygium aromaticum*), lemon (*Citrus limon*), and garlic (*Allium sativum*) (Table 3).

To achieve herd immunity through mass immunization programs and the pressing demand to develop effective anti-COVID-19 treatments, several pharmaceutical drugs have been repurposed to treat COIVD-19 including hydroxychloroquine, lopinavir/ritonavir/darunavir/umifenovir, remdesivir, and favipiravir [15]. Recently, Pfizer's Paxlovid, made up of both nirmatrelvir and ritonavir and oral tablets, has been granted an emergency use authorization (EUA), by the U.S. Food and Drug Administration (USFDA), for the treatment of COVID-19 in both adults and children (USFDA, 2022). However, to be effective, these compounds would need to be taken at relatively great continuous doses. Therefore, they could have inherent toxic potencies. For this reason, natural products from medicinal plants hold promise [2, 16, 17].

Since the beginning of the COVID-19 outbreak, traditional herbal remedies have been employed. Of note, 90% of 214 patients treated in China recovered after using some of these traditional treatments. Moreover, natural remedies, based on honey, seed oil of black cumin (*Nigella sativa*), and flowers and buds of chamomile (*Anthemis hyaline*) have been reported to be effective against COVID-19 treatment in the Middle Eastern countries Egypt and Saudi Arabia [16]. In Africa, represented by the Democratic Republic of Congo, a remedy made up of clove (*Syzygium aromaticum*), blue gum (*Eucalyptus globulus*), lemon grass (*Cymbopogon citratus*), and ginger (*Zingiber officinale*) has been used to fight against COVID-19.

Regarding the antiviral activity of the most cited plants in our survey, Table 4 summarizes some studies of these plants against different types of viruses. By use of molecular docking, the antiviral activity of eucalyptus was determined against herpes simplex virus 1, herpes simplex virus 2 [18], rotavirus Wa strain, adenovirus type 7 [19], and SARS-CoV-2 [20, 21]. Moreover, garlic has been used for centuries in the treatment of diseases such as viral diseases. Antiviral activity of *Allium sativum* has been confirmed against several viruses such as influenza A and B [22], herpes simplex virus 1, herpes simplex virus 2 rhinovirus, and human immunodeficiency virus (HIV) [23, 24]. *Citrus limon*, which is used by the Moroccan population to treat COVID-19, is rich in flavonoids like diosmin, eriocitrin, and hesperidin, which possessed biological activities including antiviral power [25]. Antiviral activity of eugeniin extracted from the *Allium sativum* and clove has been reported to be effective against herpes by inhibiting the viral DNA polymerase, which in turn affects DNA synthesis [26]. Moreover, eugenol showed antiviral activity against human herpes simplex [27].

#### 3.2.2. Mode of Use of Plants

**Figure 1:**
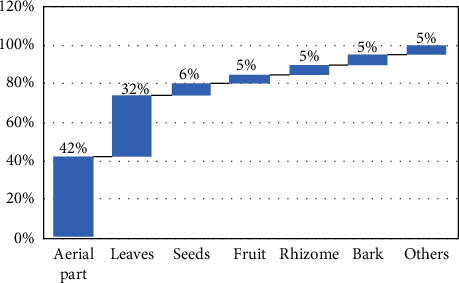
Part of plants used by the Moroccan population to prevent or treat COVID-19.

**Figure 2:**
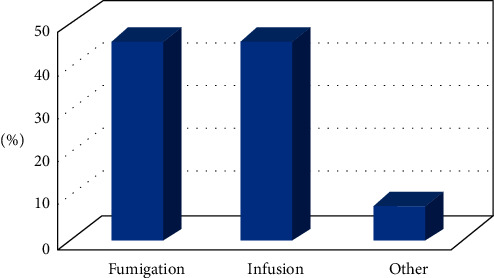
Preparation mode of plants used by the Moroccan population to prevent or treat COVID-19.

Results showed that 70% of the population use the leaves or aerial parts of plants in the treatment (Figure 1). This can be explained by the easy harvesting of aerial parts and the accessibility facilities [6]. Fumigation represents the most used method to prepare natural preparation against COVID-19, followed by infusion (Figure 2). Generally, fumigation was used in traditional medicine to treat pulmonary and neurological diseases [28]. Since the SARS-CoV-2 virus infects the respiratory system, people prefer using fumigation for treatment. Another reason why fumigation is used is that vapors can play a role in disinfestation.

**Figure 3:**
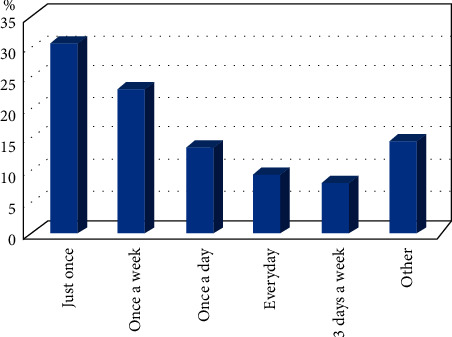
Duration of plant use by the Moroccan population during the COVID-19 pandemic.

Regarding the treatment period, Figure 3 shows that 30.6% of the population used plants at least one time from the beginning of the pandemic up to the date of investigation, 23.1% once a week, 13.9% once a day, and 9.3% every day during the outbreak.

### 3.3. Toxic Plants

The empiric use of plants for medication is always linked to risks of toxicity [29]. Our results showed that 5 out of 21 plants used by the Moroccan population for treating or preventing COVID-19 were listed to be toxic (Table 5).

Concerning the toxicity of *Artemisia herba-alba*, a study by Abderrahman and Shbailat showed harmful effects on the division of bone marrow cells and the induction of chromatid exchanges at doses of 375 and 500 *μ*g ml^−1^ [30], while another study mentioned the potential renal toxicity of this plant [31]. *N. oleander* is known for its toxicity due to the presence of cardiac glycosides in all plant parts. Cardiac glycosides inhibit Na+/Ka + ATPase pumps in cardiac cells, which lead to hyperkalemia [32–34].

*Pistacia lentiscus* is also a toxic plant whose oils cause a decrease in hepatic cytochrome P450 activity. Subacute administration of *P. lentiscus* extract in rats results in hepatic fibrosis and mild cholestasis [35–37]. Little research mentions the toxicity of *Olea europaea*. However, administration of the leaf extract of this plant for a longer period may lead to liver and kidney damage as reported in previous works [38, 39]. Furthermore, *Juniperus thurifera* oils can cause severe gastrointestinal irritation and intense congestion of the genitourinary system and intestines [40].

## 4. Conclusion

The present study documented medicinal plants used by the Moroccan population against COVID-19. The results showed that many plants used to fight the causative agents of the severe acute respiratory syndrome, including *Artemisia herba-alba* and *Nerium oleander*. Notably, antiviral activity of most reported plants has been confirmed against some viruses elsewhere, but no activity has been yet approved in in vivo studies. Some toxic pants were included in natural preparations used by the Moroccans to control SARS-CoV-2, and hence people should pay more attention to the non-approved natural products.

## Figures and Tables

**Table 1:** Distribution of the population by region.

Region	Effective
Fez-Meknes	642
Casablanca-Settat	156
Rabat-Salé-Kénitra	126
Béni Mellal-Khenifra	96
Dakhla-Oued Ed-Dahab	69
Marrakech-Safi	60
Drâa-Tafilalet	60
Laâyoune-Sakia El Hamra	54

**Table 2:** Sociodemographic characteristics.

Sociodemographic characteristics		Percentage
Gender	Men	63
	Women	37

Age (years)	<20	8.22
	20–40	77.37
	40–60	10.82
	60–80	0.77

Level of education	Primary	1.5
	Secondary	27.6
	University	70.9

Monthly income	<100 USD	41.6
	100–500 USD	24.5
	500–1000 USD	23.4
	1000–2000 USD	7.3
	>2000 USD	3.2

Locality	Urban	20
	Rural	80

Medical assistance regime (RAMED)	Yes	73
	No	27

USD: United States dollar.

**Table 3:** Ethnobotanical data.

Name of plant	Families	Vernacular name	Part of plant	Use mode	NTC
*Eucalyptus*	Myrtaceae	Eucalyptus	Leaves	Fumigation	495
*Syzygium aromaticum*	Myrtaceae	Krenfel	Cloves	Fumigation	369
				Infusion	
*Allium sativum*	Amaryllidaceae	Touma	Garlic	Raw	225
*Citrus limon*	Rutaceae	Limon	Fruit	Juice	270
*Lavandula officinalis*	Lamiaceae	Khzama	Aerial part	Infusion	72
				Fumigation	
*Trigonella foenum-graecum*	Fabaceae	Helba	Seeds	Infusion	54
*Mentha pulegium*	Lamiaceae	Flio	Aerial part	Infusion	63
				Fumigation	
*Rosmarinus officinalis*	Lamiaceae	Azir	Aerial part	Infusion	45
				Fumigation	
*Artemisia herba-alba*	Asteraceae	Chih	Aerial part	Fumigation	117
				Infusion	
*Cinnamomum verum*	Lauraceae	Karfa	Dried bark	Infusion	72
*Juniperus thurifera*	Cupressaceae	Arar	Aerial part	Fumigation	45
Nerium oleander	Apocynaceae	Defla	Leaves	Fumigation	27
*Pistacia lentiscus*	Anacardiaceae	Drou	Leaves	Fumigation	9
*Aloysia triphylla*	Verbenaceae	Lwiza	Leaves	Infusion	9
*Mentha rotundifolia*	Lamiaceae	Marsita	Aerial part	Infusion	9
*Laurus nobilis*	Lauraceae	Moussa	Leaves	Fumigation	18
*Ocimum basilicum*	Lamiaceae	Ri7an	Aerial part	Infusion	9
				Fumigation	
*Thymus vulgaris*	Lamiaceae	Zaater	Aerial part	Infusion	126
*Zingiber officinale*	Zingiberaceae	Zanajbil	Rhizome	Infusion	45
*Olea europaea*	Oleaceae	Zitoun	Leaves	Fumigation	46

NTC: total number of citations.

**Table 4:** Antiviral activity of the most cited plants in our survey against different types of viruses.

	Herpes simplex virus 1	Herpes simplex virus 2	Rotavirus Wa strain	Adenovirus type 7	SARS-CoV-2	Influenza A and B	HIV
*Eucalyptus*	+	+	+	+	+*∗*	−	−
*Allium sativum*	+	+	−	−	−	+	+
*Citrus limon*	+	+	−	−	−	−	−

+: effective against virus; −: no effect of plant against the virus; ^∗^study carried out by molecular docking. HIV: human immunodeficiency virus.

**Table 5:** Toxic plants used by the Moroccan population in the prevention or treatment of COVID-19.

Scientific name	Families	Vernacular name	Part used
*Artemisia herba-alba*	Asteraceae	Chih	Aerial part
Nerium oleander	Apocynaceae	Defla	Leaves
*Pistacia lentiscus*	Anacardiaceae	Drou	Leaves
*Olea europaea*	Oleaceae	Zitoun	Leaves
*Juniperusthurifera*	Cupressaceae	Arar	Aerial part

## Data Availability

The data used to support the findings of this study are included within the article.
